# Hidden Diversity in the Iberá Wetlands: Fern and Lycophyte Richness and Biogeographic Boundaries

**DOI:** 10.3390/plants15030378

**Published:** 2026-01-26

**Authors:** Esteban Ismael Meza-Torres, Federico Carlos Arias, Patricia Estefania Meza-Torres, Saúl Páez, Hector Alejandro Keller, Michael Kessler

**Affiliations:** 1 Unidad Ejecutora Lillo, Fundación Miguel Lillo—Consejo Nacional de Investigaciones Científicas y Técnicas (CONICET), Tucumán T4000JFD, Argentina; 2 Instituto de Investigación para el Desarrollo Territorial y del Hábitat Humano, Universidad Nacional del Nordeste—Consejo Nacional de Investigaciones Científicas y Técnicas, Resistencia 3500, Argentina; fedecarlosarias222@gmail.com (F.C.A.); pmezatorres@conicet.gov.ar (P.E.M.-T.); 3 Facultad de Humanidades, Universidad Nacional del Nordeste, Resistencia 3500, Argentina; 4 Instituto de Botánica del Nordeste, Universidad Nacional del Nordeste—Consejo Nacional de Investigaciones Científicas y Técnicas (CONICET), Corrientes 3400, Argentina; saulpaez@outlook.com (S.P.);; 5 Department of Systematic and Evolutionary Botany, University of Zurich, 8001 Zürich, Switzerland

**Keywords:** Iberá Wetlands, Ituzaingó–La Paz fracture, fern diversity, lycophytes, biogeographic boundaries, Paranaense and Chaco provinces

## Abstract

The Iberá Wetlands in northeastern Argentina constitute the second largest wetland system in South America, yet the fern and lycophyte flora of this region remains poorly documented. The aims of this work were to update the species richness of these plant groups, evaluate the intensity of collecting efforts, identify conservation priorities, estimate the potential true species richness, and make biogeographical inferences. We compiled a database of species from multiple sources, and the study area (21,853 km^2^) was divided into 19 grid cells for analysis. Sampling effort and species richness were quantified, and non-parametric estimators (Chao2, ICE, Jack2) were used to evaluate inventory completeness. Several similarity analyses were performed using the Jaccard index, incorporating reference areas from the Chaco and Paranaense phytogeographic provinces. The Ituzaingó–La Paz geological fracture and the geological formations present in the area were also considered. We recorded 76 taxa, whereas estimators suggested a potential richness of 130–140 species. The center of the Iberá Wetlands showed the lowest sampling effort, while the eastern sector exhibited the highest species richness. The distribution of species appears to be correlated with geological formations. These findings emphasize the importance of continuing sampling in the area.

## 1. Introduction

The “Esteros del Iberá”, located in northeastern Argentina, is the second largest wetland in South America. The vast expanse and limited accessibility of this subtropical wetland have long hampered the study of the lacustrine system [1]. Several conservation and restoration projects are being carried out in this region, and it is currently the focus of sustainable use initiatives promoted by the government, such as ecotourism [2].

Arbo and Tressens [3] edited an ecological flora of the Iberá wetlands that includes a detailed checklist of vascular plants. They listed a total of 44 species of lycophytes and ferns in a study area close to 12,000 km^2^ [3,4]. Subsequently, the execution of multiple research projects intensified the botanical collections in this area, but the list of fern species has not been updated. Given this background, two key questions arise: How complete are the existing fern and lycophyte collections from this area? And what is the actual species richness of these plant groups in the Iberá Wetlands?

**Figure 1:**
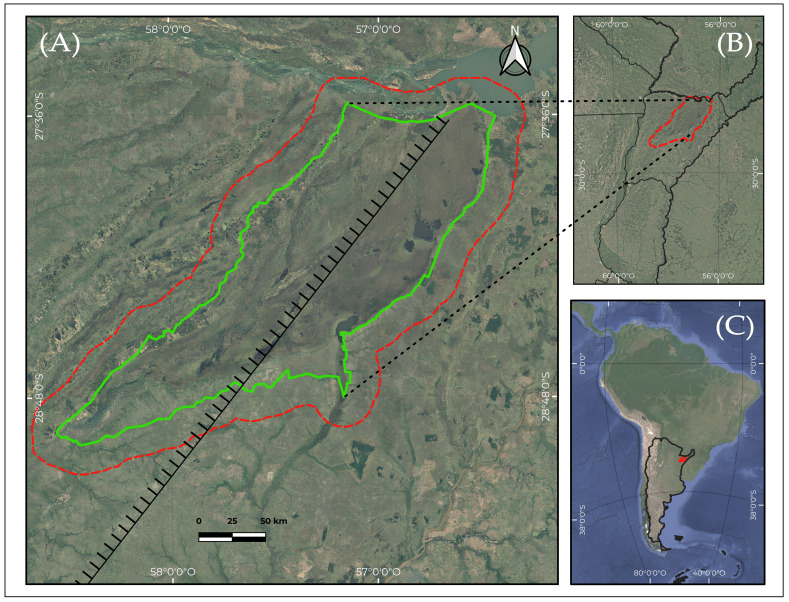
(**A**) Location of the study area within the Iberá Macrosystem, Corrientes Province, Argentina. The legally protected area (Iberá Provincial and National Parks plus private reserves) is outlined in green. The total study area, including the 14-km buffer zone, is delineated in red. The comb line represents the Ituzaingó–La Paz fracture proposed by Iriondo [5], with the teeth indicating the more depressed sector. (**B**) Location of the reserves used for the cluster analyses: 1, Reserva Educativa Colonia Benítez; 2, Reserva del Valle de Cuñá Pirú; 3, Reserva de Uso Múltiple Guaraní; 4, Parque Provincial Teyú Cuaré. (**C**) Location of the study area within Argentina.

**Figure 2:**
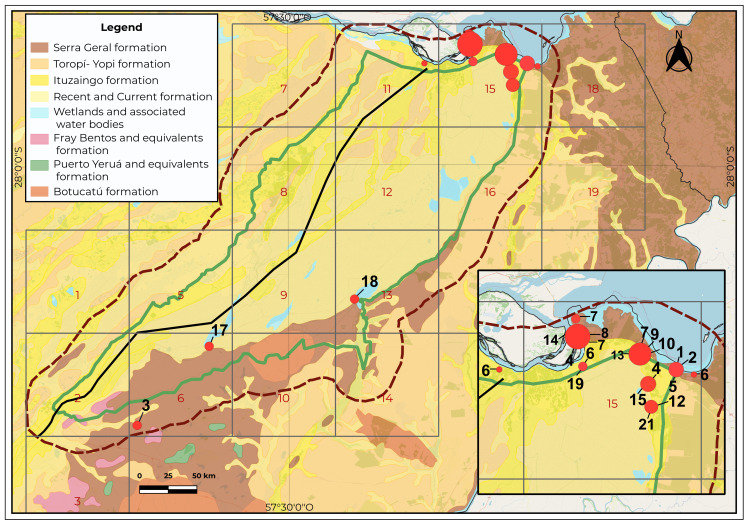
Location of species restricted to the eastern sector relative to the Ituzaingó–La Paz fracture in the Iberá Macrosystem (Corrientes Province, Argentina). The legally protected areas (Iberá Provincial and National Parks plus private reserves) are outlined in green. The total study area, including the 14-km buffer zone, is delineated with a dashed brown line. The Iberá Wetlands are divided by the line of greatest depressions, representing the hypothetical Ituzaingó–La Paz geological fracture (black line). The geological formations that outcrop within the study area are shown in different colors. The distribution of the 24 fern and lycophyte species exclusive to the eastern sector is depicted as hotspots based on taxon concentration (with their reference numbers shown in black). Burgundy numbers indicate the cell numbers. The complete list of species, including their reference numbers, is provided in Section 2.

Further, we do not have documentation of the spatial variation of fern species richness and community composition within the Iberá region. This is particularly interesting because the wetlands lie along two important geological and biogeographical transition zones. One is the Ituzaingó–La Paz geological fracture (Figure 1), which runs across Corrientes Province from northeast to southwest, roughly dividing it into two equal parts. This fracture has a total length of about 480 km and then extends to the SW along the Paraná River [5]. This fracture is inferred in the subsurface (with general consensus) based on the pattern and drainage density of the surface runoff network (O. Orfeo, pers. comm.). It has been present since at least the Pliocene and marks the boundary between the Paraná River basin and the Cretaceous basalt region. To place this structural feature in context, it is important to summarize the main geological units underlying the Iberá region. This macrosystem is underlain by six geological formations. The most prominent ones exposed at the surface within the study area are Botucatú Formation, of Jurassic age, in southeastern of study area; basaltic outcrops of the Serra Geral Formation (Lower Cretaceous) in the southeastern and far northeastern sectors; the Fray Bentos Formation, of Paleogene–Oligocene age, cropping out in a small area of the southwestern sector; and sediments of the Ituzaingó Formation (Pliocene) exposed along the northern and western flanks, with additional isolated outcrops toward the central area. The Toropí–Yupoí Formation (Late Pleistocene) borders large portions of the eastern and western flanks. The central Iberá region is dominated by recent lacustrine and palustrine deposits [6,7] (Figure 2).

On the other hand, the Iberá wetlands are located on the border between the Paranaense phytogeographic province in the East and Chaco phytogeographic province in the West, constituting an ecotone region [8]. The boundary between these two phytogeographic provinces has been described as diffuse, with its placement varying among authors [8,9,10]. This raises the question of whether the boundaries between the phytogeographical provinces present in the study area correlate with the fracture line or with the different geological formations and their associated lithological characteristics. Ferns and lycophytes are suitable for this biogeographic analysis because their efficient spore dispersal by wind does not depend on animal vectors, so that their distributions are considered to be more closely aligned with environmental conditions, especially soil and climate characteristics [11,12,13,14].

The Iberá wetlands are under increasing threat from climate change. In northern Argentina, the La Niña effect caused a drought episode in 2019–2022, which is among the five worst droughts recorded in the region since the 1950s [15]. In particular, the greater Iberá region was affected by an extraordinary water and fire emergency. During 2022, the accumulated precipitation ranged between 700 mm and 800 mm depending on the location, which was about half of the long-term annual mean of about 1600 mm [16]. This led to the occurrence of megafires, that is, fires that exceeded 10,000 ha [17,18], which towards the end of February 2022 covered an area of more than one million hectares within the provincial territory [19]. These megafires produced a significant loss in biodiversity, which have been studied in vertebrate animals [16]. However, there are still no exhaustive studies on the impacts on vegetation.

Taking into account the conservation value of this area, the risk of future megafires, and its particular phytogeographic setting, the objectives of this study were to (a) update the species richness of ferns and lycophytes relative to the checklist of Arbo and Tressens [3]; (b) evaluate the intensity of collecting efforts across different sectors of the protected area; (c) analyze the distribution of fern and lycophyte diversity within the Iberá Wetlands to identify conservation priorities; (d) estimate the potential true species richness of these groups in the study area; and (e) make biogeographical inferences in relation to its ecotonal position and its lithological diversity.

## 2. Results

### 2.1. Sampling Effort, Species Richness Pattern*s*

A complete database with 379 specimens of the fern and lycophyte taxa recorded in the Iberá Wetlands, including voucher information and geographic coordinates, is provided in Table S1 (Supplementary Material).

A total of 22 families were recorded in the Iberá Macrosystem, comprising 48 genera and 76 infrageneric taxa (75 species plus one species represented by two varieties). The richest families were Pteridaceae (9 genera, 16 species), Polypodiaceae (4 genera, 7 species), Thelypteridaceae (5 genera, 7 species), and Salviniaceae (2 genera, 6 species). Other families showed lower diversity, including Lycopodiaceae (3 genera, 4 species), Blechnaceae (4 genera, 4 species), Aspleniaceae (1 genus, 4 species), Ophioglossaceae (1 genus, 3 species), Isoëtaceae (1 genus, 2 species), Selaginellaceae (1 genus, 2 species), Hymenophyllaceae (1 genus, 2 species), Gleicheniaceae (2 genera, 2 species), Osmundaceae (2 genera, 2 species), and Marsileaceae (2 genera, 2 species), whereas Dennstaedtiaceae, Equisetaceae, Lygodiaceae, Lindsaeaceae, Psilotaceae, and Cyatheaceae were represented by a single species each; Anemiaceae was represented by two species and one variety. The best-represented genera were *Doryopteris*, *Salvinia*, and *Asplenium* (four species each), followed by *Adiantopsis*, *Anemia*, *Christella*, and *Ophioglossum* (three species each), whereas *Isoëtes*, *Lycopodiella*, *Selaginella*, *Trichomanes*, *Pityrogramma*, *Pecluma*, *Pleopeltis*, *Microgramma*, and *Azolla* were represented by two species. The remaining 32 genera are each represented by a single species. The full list of recorded taxa, following the taxonomic circumscription of the PPG I [20], is provided in Appendix A.

**Figure 3:**
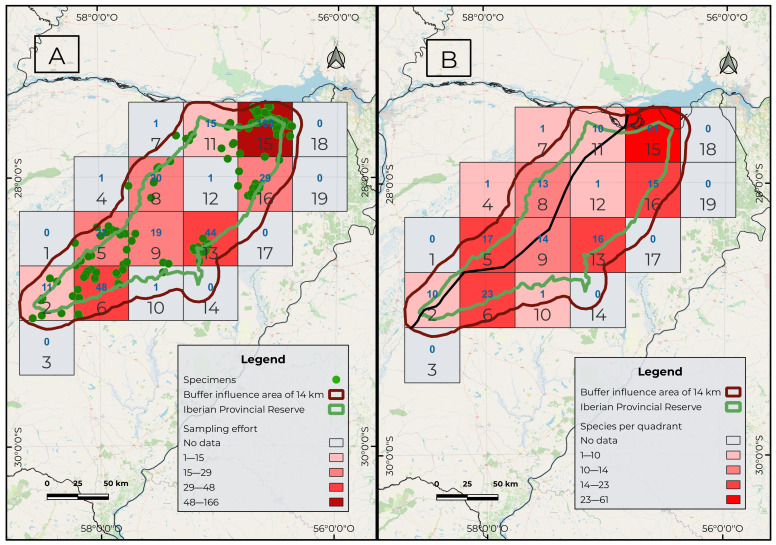
Study area divided into a grid of 19 cells, each 45 km per side. The black line represents the Ituzaingó–La Paz fracture proposed by Iriondo [5], reconstructed here by connecting the lowest elevation points in the area. Spatial pattern analysis of lycophyte and fern records in the Iberá Wetlands: (**A**) sampling effort represented by the number of specimen records per cell, and (**B**) species richness expressed as the number of species per cell. Cell numbers are shown in black, and absolute values per cell in blue.

The analyses revealed varying levels of sampling effort across the grids (Figure 3A). Grid cell No. 15 of NW Ituzaingó showed the highest number, with 166 specimens, followed by cell 6 of Mercedes, with 48 specimens, and cell 13 of Carlos Pellegrini, with 44. The remaining cells showed values ranging from 1 to 29 specimens.

Species richness per grid cell was also highest in cell 15, with 61 species (Figure 3B). In decreasing order of richness, the following were recorded: cell 6 (23 spp.), cell 5, corresponding to the town of Concepción (17 spp.), and cell 13 (16 spp.). In the remaining cells, values ranged from 1 to 15 species.

**Figure 4:**
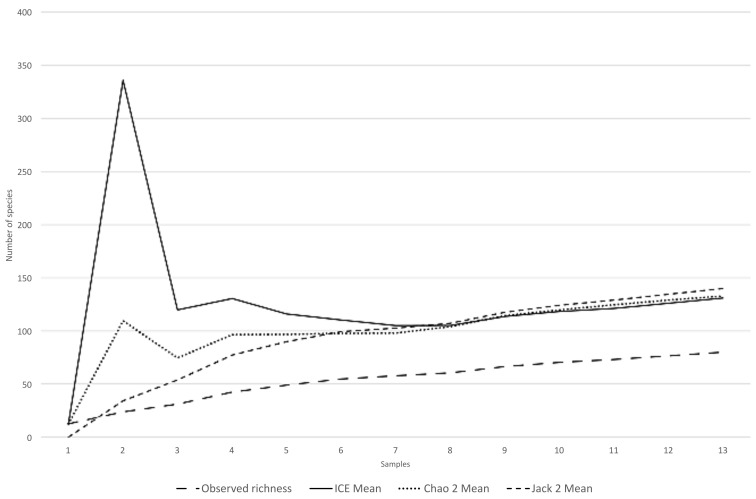
Species accumulation curve for lycophytes and ferns in the Iberá Wetlands, showing observed richness and estimates from Chao2, ICE, and Jack2.

The non-parametric estimators yielded values of estimated total species richness of 132 species (Chao2), 130 species (ICE), and 139 species (Jack2) (Figure 4).

### 2.2. Biogeographical Patterns and Floristic Affinities

The reconstructed Ituzaingó–La Paz fracture line started east of Villa Olivari, extended southwestward, and merged with the course of the Corriente River (Figure 2 and Figure 3A,B). The western sector did not contain any exclusive species. All taxa recorded in the western portion showed broad distributions extending into the eastern sector and further eastward into Misiones and Brazil. Filtering the species restricted to the eastern side of this hypothesized line yielded 24 taxa, whose occurrences are shown in Figure 2: 1. *Adiantopsis radiata*, 2. *Asplenium inaequilaterale*, 3. *A. ulbrichtii*, 4. *Blechnum occidentale*, 5. *Ctenitis submarginalis*, 6. *Cyathea atrovirens*, 7. *Cyathea delgadoi*, 8. *Dicranopteris flexuosa*, 9. *Didymochlaena truncatula*, 10. *Doryopteris arifolia*, 11. *D. triphylla*, 12. *Lindsaea divaricata*, 13. *Lomariocycas schomburghii*, 14. *Lygodium volubile*, 15. *Lytoneuron lomariaceum*, 16. *Megalastrum connexum*, 17. *Microgramma squamulosa*, 18. *Osmundastrum cinnamomeum*, 19. *Regnellidium diphyllum*, 20. *Sticherus lanuginosus*, 21. *Trichomanes cristatum*, 22. *T. pellucens*, 23. *Vittaria lineata*, and 24. *Selaginella marginata*. The highest concentration of *Cyathea atrovirens* specimens occurred in cell 15, located east of the hypothetical fracture line. However, a single population was found only a few kilometers west of this line, which represents the westernmost limit of the species distribution in Argentina. Given the strongly eastern distribution pattern and the fact that the western record corresponds to an isolated marginal occurrence, we considered *C. atrovirens* as exclusive to the eastern sector.

In the Jaccard analysis comparing the eastern and western sectors of the Iberá Wetlands we observed a moderate floristic dissimilarity (Jaccard = 0.38).

**Figure 5:**
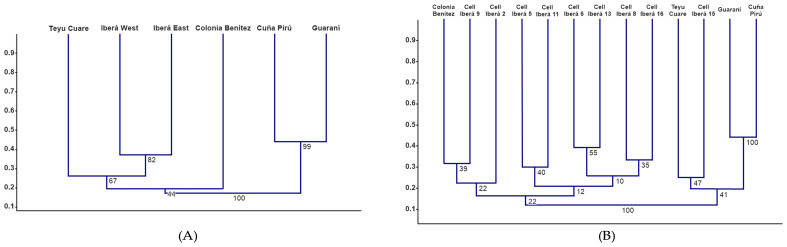
Cluster analyses (UPGMA, Jaccard index) based on the lycophyte and fern species recorded in the Iberá Wetlands and reference areas. Bootstrap values (1000 replicates) are shown above the branches. (**A**) Similarity relationships among the eastern and western sectors of the Iberá Wetlands and the protected areas of the Paranaense phytogeographic province (Teyú Cuaré, Cuña Pirú, Guaraní natural reserves) and Chaco (Colonia Benítez natural reserve) phytogeographic provinces. (**B**) Cluster analysis of the Iberá grid cells containing more than five species, compared with the same external reference areas.

The expanded database, including the reference areas from the Chaco and Paranaense biogeographical provinces, comprised 152 infrageneric taxa. Regarding the comparison of both Iberá sectors with the surrounding protected areas, Iberá West and Iberá East first clustered together (bootstrap = 82) (Figure 5A). This Iberá group then joined Teyú Cuaré, the westernmost site of the Paranaense Province, although with low support (bootstrap = 67). Finally, the whole cluster grouped with Colonia Benítez (Chaco Province) with very low support (bootstrap = 44), indicating that these broader associations are not statistically robust.

The cells that recorded five or more species were 2, 5, 6, 8, 9, 11, 13, 15, and 16. The cluster analysis including the eight Iberá cells with more than five species and the external reference areas showed mostly weakly supported patterns, without a clear floristic structure among Iberá sites (Figure 5B). Within Iberá, the association between Cells 6 and 13 displayed the highest internal support (bootstrap 55), while the remaining nodes showed low bootstrap values and did not reveal a consistent geographic or ecological pattern. The Cell 15 was positioned closest to the Paranaense reference areas (cell 15 and Teyú Cuaré; bootstrap 47).

## 3. Discussion

The sampling effort map showed that grid 15 (Figure 3A) had the highest sampling intensity. This result is consistent with the recent intensive floristic surveys conducted in areas adjacent to the Yacyretá Binational Entity [20]. The second and third grids with the highest sampling effort were those encompassing the towns of Mercedes (cell 6) and Colonia Carlos Pellegrini (cell 13). This pattern was expected, as both towns provide the most developed accommodation facilities and have historically served as logistical bases for research projects conducted in the area. On the other hand, the central region of the study area (cells 9 and 12; Figure 3A) showed the lowest sampling effort. These areas correspond to lagoons and ‘embalsados’—floating vegetation islands with variable surfaces. Access to these environments is very difficult and must be made by small boat. In such habitats, it is likely that records are still pending for marsh or floating species of lycophytes and ferns, such as those belonging to the genera *Isoëtes*, *Ceratopteris*, and *Salvinia*, or species of Thelypteridaceae or Osmundaceae

Species richness patterns showed a certain degree of parallelism with the distribution of sampling effort. This study documented 76 infrageneric taxa, whereas Arbo and Tressens [3] had previously cited only 44 for the area, highlighting a substantial increase in the known diversity. The non-parametric estimators suggest that the Iberá Wetlands may harbor between 130 and 140 fern and lycophyte species, i.e., only 60% of the estimated richness has been recorded so far. Consequently, even the areas identified as richest in this study are probably under-sampled and their species inventories remain incomplete. In Corrientes Province, around 105 pteridophyte taxa are known [4,20,21,22,23,24,25], which indicates that the Iberá Wetlands could concentrate most of the provincial diversity. Considering that the total richness of Argentina is about 430 species [25,26,27,28,29,30], if our estimates are correct, the Iberá Wetlands alone may harbor nearly one third of the national fern and lycophyte diversity.

The cluster analysis of the two areas in relation to the hypothetical fracture reflects a moderate floristic dissimilarity between the eastern and western sectors (Jaccard distance ≈ 0.37). This indicates that both sectors differ to some extent in species composition, although not enough to support a deep biogeographic break across the hypothetical Ituzaingó–La Paz fracture. This pattern is largely due to the fact that the western sector does not contain exclusive species. All taxa recorded there are widely distributed and also occur east of the fracture line. For this reason, the dendrogram provides only limited structural information.

Similarity analyses between the two Iberá sectors and the external reference areas showed that both Iberá East and Iberá West clustered with one Paranaense area (Teyú Cuaré) and one Chaco area (Colonia Benítez). The grouping between Iberá and Teyú Cuaré occurred at a relatively low similarity level (Jaccard distance ≈ 0.27), a pattern that has also been noted previously by Meza Torres et al. [4]. However, the bootstrap support values in this analysis were low, indicating that these groupings are not statistically robust and should be interpreted with caution.

The similarity analysis of the nine Iberá cells in relation to the external reference areas showed that cell 15 clustered with the three Paranaense reserves. This pattern is consistent with the fact that this cell contains the highest concentration of species that extend westward from the Paranaense province, forming the westernmost limit of several taxa within Argentina [20,31]. Another noteworthy pattern was the clustering of cells 6 and 13, which, despite showing only moderate support (bootstrap 55), was geographically coherent with their shared position on the Serra Geral Formation. On the other hand, the clustering of the Colonia Benítez cell with Iberá 9 and Iberá 2, as well as the grouping of Iberá 5 with Iberá 11, showed low support (bootstrap values between 22 and 40), yet the pattern becomes geographically coherent when overlaid with the map of Paraná sedimentary deposits described by Iriondo [5]. These deposits also correspond broadly with the Humid Chaco region [8]. Nevertheless, these correlations should be interpreted with caution given the low bootstrap support.

The distribution of the 24 species exclusive to the eastern sector showed a strong association with the Serra Geral Formation, which extends broadly into the Paranaense phytogeographic province. This supports the idea that the geological continuity of this formation may facilitate the presence of Paranaense taxa into the eastern margin of the Iberá wetlands.

Overall, the results of the cluster analyses suggest that the northeastern sector of the Iberá wetlands represents the westernmost limit of fern and lycophyte species characteristic of the Paranaense province. The remaining regions show stronger affinities with the Chacoan province, forming a mosaic of communities associated with edaphic heterogeneity linked to the Ituzaingó–La Paz fracture and underlying geological formations. Frederiksen et al. [32] have shown that the Paranaense forests were phytogeographically more similar to Amazonian forests some 30 million years ago, but that the formation of the South American dry diagonal during the mid-Miocene separated these humid forest domains and led to a compositional divergence of the Paranaense province. The formation of the dry regions in turn facilitated the evolution of a species poor drought adapted fern flora, which then dispersed into the Paranaense forest, creating novel phytogeographical connections. Interestingly, there was relatively stronger dispersal from dry into humid habitats than vice versa. One explanation for this is that drought-adapted species may more easily find dry microhabitats in humid regions, especially if they are epiphytic. In contrast, dry regions only offer few suitable microhabitats for humid-adapted species [33].

Our reconstruction of the Ituzaingó–La Paz fracture line, which starts east of Villa Olivari and extends south-westwards to merge with the course of the Corriente River, broadly separates the major soil types. On the western side lie sandy hills [34], corresponding to the area influenced by the Paraná River, which has shaped this region since the Pliocene. During the Pliocene and Pleistocene, the river deposited its sedimentary load, giving rise to the Ituzaingó Formation. This formation is characterized by fine, ochre-coloured quartz sands interbedded with silts [8]. The species that fail to cross the Iberá depression are mostly distributed in ferruginous soils, reddish in varying intensity, underlain by detrital subsoils of similar composition and color, except for the presence of rock fragments and intercalated concretionary limonite layers, or on solid eruptive rocks, partly tuffaceous, derived from diabase–basaltic magma of Triassic age. The associated ferns species occur in northeastern Argentina, where they are characteristic of the Paranaense phytogeographic province and extend into Brazil to varying degrees. It should be noted that *Asplenium inaequilaterale*, *Blechnum occidentale*, *Ctenitis submarginalis*, *Doryopteris triphylla*, and *Microgramma squamulosa* have disjunct distributions, reoccurring in the Yungas forests of northwestern Argentina [35,36,37,38,39].

On the other hand, *A. ulbrichtii* and *M. squamulosa* extend southwards as far as Buenos Aires Province, but in Corrientes do not cross the depression line of the Iberá. Here we can observe that, within only a few kilometers, there is an abrupt change in soils, while climatic characteristics remain relatively stable. In this context, the differences in soils on both sides of the Iberá marshes could act as a vicariant barrier. This interpretation appears consistent with the conclusions reached for Amazonian rainforests by Tuomisto et al. [11]. These authors highlighted niche-related species sorting as an important process shaping species co-occurrence, turnover, and richness patterns within rainforests. They observed that all *Adiantum* and *Lindsaea* species, including the most abundant ones, had sufficiently narrow realized niches to serve as reliable indicators of edaphic and floristic variation within the rainforest.

## 4. Materials and Methods

### 4.1. Study Area and Spatial Framework

The Iberá Wetlands are located in the central region of Corrientes Province, Argentina. The study area was delimited using the current protected areas of the Iberá Wetlands, plus an additional area of influence of 14 km (Figure 1A, Figure 2 and Figure 3). Their geographic center is situated at 28.287° S and 57.371° W. In this way, we sought to include the regions affected by the maximum flood levels of the wetlands. The total study area encompassed 21,853 km^2^ and was partitioned into 19 grid cells, each measuring 45 km per side (Figure 3A,B). The climate in this wetland is humid subtropical and presents differences between localities located in the extreme North and South. The monthly average minimum temperature, recorded in June and July, is 16–17 °C. The absolute minimum is −2 °C, and there is a low frequency of annual frosts. The maximum temperature average is recorded in January and February and is 27 and 28 °C. The absolute maxima reach 44 °C [40]. Average annual precipitation in the province is about 1600 mm [16].

The study area encompasses six geological formations. The oldest formation corresponds to Botucatú Formation, of Jurassic age, which crops out as an oval-shaped exposure along the southeastern boundary of the study area. It consists of compact sandstones with medium- to fine-grained particles composed mainly of well-rounded quartz grains (90% by weight), displaying low-angle planar or cross stratification. Its color ranges from pink to reddish. Locally, the unit includes silicified intervals and polymictic conglomeratic layers at its base. The Serra Geral formation (Lower Cretaceous), characterized by outcrops of black, grayish, and reddish basalts of augitic composition, was formed by successive lava flows. The main soils of this region belong to the alfisol, mollisol, ultisol, and oxisol orders, and extend widely into the province of Misiones. The Fray Bentos Formation, of Paleogene–Oligocene age, crops out in a small area of the southwestern sector and is composed of pink siltstones with calcium carbonate content. Within the study area, the Ituzaingó Formation is exposed as medium- to fine-grained sandstones, yellowish to ochre in color, with interbedded grayish-green silt-clay layers and occasional gravel. It also contains a clay fraction dominated by kaolinite, followed by montmorillonite. This formation is frequently overlain by Quaternary palustrine deposits. The Toropí–Yupoí Formation (Pleistocene) is characterized by silts, plastic clays, and fine sands. Finally, the Recent and modern deposits consist of slightly clayey silts, sands, granules, and gravel [6,7].

The Iberá macrosystem comprises a mosaic of vegetation formations, including [34,41]: (1) aquatic communities dominated by submerged and floating species such as *Cabomba caroliniana*, *Ceratophyllum demersum*, *Egeria najas*, *Eichhornia crassipes*, *Salvinia* spp., and *Azolla* spp. (2) Amphibious communities, represented by extensive stands of *Cyperus giganteus*, *Rhynchospora corymbosa*, *Schoenoplectus californicus*, *Zizaniopsis bonariensis*, *Typha* spp., and *Thalia* spp. (3) Tall grasslands (“pajonales”), dominated by tall hygrophilous grasses such as *Coleataenia prionitis*, *Paspalum durifolium*, *Hymenachne grumosa*, *Sporobolus spartinae*. (4) Weed-dominated patches (“malezales”), composed of herbaceous vegetation that develops in semi-permanent bodies of water, where three complexes alternate—aquatic, marshy, and terrestrial—depending on water availability. When water is abundant, *Azolla*, *Salvinia*, and *Lemna* are presents; as water availability decreases, meadows *of Luziola peruviana* form. As water levels drop further, stands of *Paspalum acuminatum*, *Paspalum modestum*, and various species of Cyperaceae develop. (5) Palustrine meadows, mainly composed of *Eleocharis montana*, *Luziola peruviana*, and *Leersia hexandra*. (6) Grasslands dominated by *Anatherum laterale*, *Axonopus fissifolius*, *Elionurus muticus* or *Paspalum notatum*. (7) Palm groves of *Copernicia alba* and *Butia* (*B. paraguariensis* and *B. yatay*). (8) Flooded and upland forests, including stands on sandy hills with *Cordia americana*, *Ficus luschnathiana*, and *Myrcianthes pungens*, as well as flood forests with *Calophyllum brasiliense* and *Nectandra angustifolia*. (9) Savannas dominated by *Neltuma* (*N. affinis* and *N. nigra*) and *Sapium haematospermum.*

### 4.2. Database Compilation

We compiled a database of fern and lycophyte species collected in the study area based on the checklist of the flora of Iberá [3]. Additional data were retrieved from (i) Flora Argentina (http://www.floraargentina.edu.ar/ accessed on 7 March 2022), (ii) the Virtual Herbarium CTES (http://ibone.unne.edu.ar/herbariovirtual/ accessed on 14 March 2022), (iii) a personal database of specimens newly collected by E.I.M.T. and H.A.K. up to May 2024, (iv) citations of specimens from the study area published in different papers [31,42,43,44], and (v) a review of specimens deposited in the CTES and LIL herbaria. It should be noted that in 1965 the Instituto de Botánica del Nordeste and its CTES herbarium were founded. Since then, CTES has assembled local collections, preserving material from its own researchers and duplicates from other institutions. These collections represent a significant dataset for the study area. For each record, we recorded herbarium, taxonomic family and species (*sensu* PPG I [45]), locality, geographic coordinates, and other data of lesser value for this study.

To achieve our objective of estimating species richness, we counted the total number of infrageneric taxa (species plus varieties). For the grid-cell richness analyses, we consider it valid to include taxa not identified to the species level, since they provide information under the concept of morphospecies—that is, specimens that are taxonomically distinct from all other specimens recorded within the same cell. In addition, the first author is preparing a separate taxonomic study that will provide detailed nomenclatural clarifications about the species of this area.

The geographic coordinates of 210 specimens that lacked georeference data on their labels were obtained by interpreting the descriptive locality information and verifying the most likely position using digital maps (Google Maps™ and Google Earth™). The resulting database was subsequently curated using OpenRefine 3.7.9 [46].

### 4.3. Sampling Effort, Species Richness and Statistical Analyses

Sampling effort and species richness were quantified as the number of specimens collected per grid cell, using the polygon counting geoprocess in R 4.3.1 [47] and QGIS 3.28.0 (Bialowieza) [48]. To estimate the potential true species richness in the study area, we applied the non-parametric estimators Chao2, ICE, and Jack2 [49]. Each grid was treated as a sampling unit. These estimators were calculated from species incidence in the 13 grids with available data using EstimateS 9.1 [50] to evaluate sampling completeness. Species accumulation curves were plotted in a spreadsheet to examine diversity and sampling effort.

### 4.4. Biogeographical Analysis

To test the hypothesis that the lowest line of the Iberá Wetlands could act as a species barrier, we delineated the putative fracture proposed by Iriondo [5]. This line was drawn by connecting the lowest depressions of the Iberá Macrosystem and was generated from a manually drawn KML file in Google Earth. Based on this line, the study area was divided into western and eastern sectors, and species records were mapped accordingly. Species occurring in both sectors were excluded, to obtain exclusive species lists to each subarea.

In order to analyze the phytogeographic relationships of the Iberá Wetlands, we conducted a series of cluster analyses using the Jaccard index. The analyses were performed with the Paired Group Algorithm (UPGMA) in PAST v.4.13 [51]. To assess the statistical support of the resulting clusters, we applied a bootstrap test with 1000 replicates.

For the comparative analysis with the Paranaense and Chaco phytogeographic provinces, we assembled a database that also incorporated fern and lycophyte inventories from the Guaraní [52], Teyú Cuaré [53,54], and Cuña Pirú [55] Reserves (Paranaense Province), as well as from the Colonia Benítez Reserve [56] (Chaco Province) (Figure 1B). The similarity analyses performed were as follows:

(a) Comparison of the species present in the eastern and western sectors of the Iberá Wetlands, separated by the line of greatest depressions.
(b) Comparison of the eastern and western sectors of the Iberá Wetlands with the four reserves from the Paranaense Province and the area from the Chaco Province.
(c) Comparison of Iberá cells with the Paranaense and Chaco areas mentioned above. We included only those cells containing more than five species, in order to avoid the instability generated by extremely species-poor units, which tend to produce unreliable similarity values and artificial clustering patterns.

To examine the relationship between species and lithology, the taxa exclusive to the eastern sector were mapped onto the Geological Map of Argentina, whose layers can be downloaded from the SEGEMAR (IGRM—Unidad de Sensores Remotos y SIG) website [57].

## 5. Conclusions

The inventory of infrageneric taxa recorded in the study area has increased by 73% compared to the checklist published by Arbo and Tressens [3]. However, the analyses show that the sampling is still insufficient to record all the taxa present, and this number could increase by an additional 40%. The central sectors of the study area should be prioritized for future sampling, as they remain the least explored due to their difficult accessibility.

The areas with the highest concentration of recorded species were located in the eastern sector, particularly in the northeastern and southeastern regions.

Because the sampling effort is still insufficient to provide a reliable estimate of species richness, robust biogeographical inferences cannot be made. However, some observations of a strong association between species distributions and edaphic heterogeneity, together with the evidence of a possible vicariant barrier linked to the Iberá depression, highlight the importance of soil-related processes in shaping floristic patterns at a regional scale. These findings not only expand the knowledge about the lycophyte and fern flora of north-eastern Argentina, but also emphasize the need to strengthen conservation and monitoring efforts in this wetland system, particularly in the face of increasing environmental threats such as wildfires and land-use change. Finally, continued field collections are essential to verify whether the estimated potential richness is accurate and to refine future biodiversity assessments.

## Data Availability

The original contributions presented in this study are included in the article/Supplementary Material. Further inquiries can be directed to the corresponding author.

## Supplementary Materials

The following supporting information can be downloaded at: https://www.mdpi.com/article/10.3390/plants15030378/s1, Table S1: Complete checklist of fern and lycophyte taxa recorded in the Iberá Wetlands, including voucher data, geographic coordinates, grid cell occurrence, and references. All references of the supplementary file (TS1) were cited in Materials and methods in the following paragraph: “(iv) citations of specimens from the study area published in different papers [31,42,43,44]”.

## Appendix A

Checklist of recorded families, genera, and species in the Iberá macrosystem. Only one reference specimen is cited; vouchers that do not specify a reference herbarium are deposited in the CTES herbarium.

ISOËTACEAE

1. *Isoëtes ekmanii* U. Weber, Depto. Concepción, Carambola, Estancia Fortín del Iberá, 17.09.1986, Pedersen 14586.
2. *Isoëtes gardneriana* Meet., Depto. Ituzaingó, Reserva Natural Mbaracaya, 27°29′07″ S 56°41′58″ W, 94 m s.n.m., 28.05.2024, Polentarrutti et al. 14.

LYCOPODIACEAE

3. *Lycopodiella geometra* B. Øllg. & P.G. Windisch, Depto. Ituzaingó, 27°34′57.4″ S 56°31′47.3″ W, 79 m s.m., 01.03.2013, Keller & Krauczuk 11325.
4. *Lycopodiella longipes* (Hook. & Grev.) Holub, Depto. Concepción, Estancia Tranquera de Hierro, 28°35′ S, 58°04–05′ W, 03.12.1996, Arbo et al. 6989.
5. *Palhinhaea cernua* (L.) Vasc. & Franco, Depto. Concepción, Estancia Tranquera de Hierro, 03.12.1996, Arbo et al. 6988.
6. *Pseudolycopodiella meridionalis* (Underw. & F.E. Lloyd) Holub, Depto. Concepción, Estancia Cerro Puitá, 31.12.1982, Pedersen 13481.

SELAGINELLACEAE

7. *Selaginella marginata* (Willd.) Spring, Depto. Mercedes, Yofre, entrada a la Estancia Mauvecin, 23.08.1984, Fernández 1017.
8. *Selaginella sellowii* Hieron. Depto. Mercedes, Felipe Yofre, Ea. La Aurora, 29°1′32″ S 58°29′22″ W, 53 m.s.n.m, 09.02.2008, Meza Torres et al. 701.

ASPLENIACEAE

9. *Asplenium inaequilaterale* Willd., Depto. Ituzaingó, Puerto Valle, Predio Forestal Pomera, Reserva interna, 25.02.2023, Meza Torres et al. 1742.
10. *Asplenium otites* Link, Depto. Ituzaingó, Puerto Valle, 01.1990, Heinonen et al. 89.
11. *Asplenium palmeri* Maxon, Depto. Mercedes, Arroyo Pay Ubre Grande, 80 m s.m., 09.02.2008, Meza Torres et al. 706.
12. *Asplenium ulbrichtii* Rosenst., Depto. Mercedes, Arroyo Pay Ubre Grande, 02.09.2008, Meza Torres et al. 706.

BLECHNACEAE

13. *Blechnum occidentale* L., Depto. Ituzaingó, Rincón de Santa María, 27°30′55′ S 56°40′03″ W, 83 m s.m., 28.04.2022, Keller et al. 14906.
14. *Lomariocycas schomburghii* (Klotzsch) Gasper & A.R. Sm., Depto. Ituzaingó, Puerto Valle, Establecimiento Puerto Valle 65 m s.m., 12.02.2016, Meza Torres et al. 1600.
15. *Neoblechnum brasiliense* (Desv.) Gasper & V.A.O. Dittrich, Depto. San Miguel, Ea. Curuzú Laurel, 14.06.1974, Schinini & González 9342.
16. *Telmatoblechnum serrulatum* (Rich.) Perrie, D.J. Ohlsen & Brownsey, Depto. Concepción, acceso hacia estancia “El Tránsito″, 28°24′13.1″ S 57°51′11.1″ W, 69 m s.m., s.f., Keller et al. 10902.

CYATHEACEAE

17. *Cyathea atrovirens* (Langsd. & Fisch.) Domin, Depto. Ituzaingó, Puerto Valle, Esteros del Iberá, Empresa Pomera, 2.03.2013, Krauczuk & Keller 99.
18. *Cyathea delgadoi* Sternb., Depto. Ituzaingo, Puerto Valle, 27°34′54.6″ S 56°31′52.6″ W, 72 m s.m., 24.05.2013, Keller et al. 11400.

DENNSTAEDTIACEAE

19. *Pteridium esculentum* (G. Forst.) Cockayne subsp. *arachnoideum* (Kaulf.) Thomson var. *arachnoideum*, Depto. San Miguel, 12 km al NE de San Miguel, Ea. Curuzú-Laurel, 27.04.1975, Schinini et al. 11441.

DRYOPTERIDACEAE

20. *Didymochlaena truncatula* (Sw.) Sm., Depto. Ituzaingó, Puerto Valle, 27°34′55.4″ S 56°31′50.5″ W, 83 m s.m., 24.05.2013, Keller et al. 11399.
21. *Megalastrum connexum* (Kaulf.) A.R. Sm. & R.C. Moran, Depto. Ituzaingó, Puerto Valle, 27°34′54.6″ S 56°31′52.6″ W, 72 m. s.m., 24.04.2013, Keller et al. 11402.
22. *Rumhora adiantiformis* (G. Forst.) Ching, Depto. Ituzaingó, Puerto Valle, 27°37′39.1″ S 56°30′02.3″ W, 101 m s.m., 10.06.2013, Keller et al. 11484.

EQUISETACEAE

23. *Equisetum giganteum* L., Depto. Ituzaingó, Rincón del Rosario, aprox. 15 Km S de Ituzaingó, 23.05.1982, Carnevali 5373.

GLEICHENIACEAE

24. *Dicranopteris flexuosa* (Schrad.) Underw., Depto. Ituzaingó, Rincón de Santa María, 27°30′55″ S 56°40′03″ W, 83 m s.m., 28.04.2022, Keller et al. 14915.
25. *Sticherus lanuginosus* (Fée) Nakai, Depto. Ituzaingó, Rincón de Santa María, 27°30′55″ S 56°40′03″ W, 83 m s.m., 28.04.2022, Keller et al. 14916.

HYMENOPHYLLACEAE

26. *Trichomanes cristatum* Kaulf. Depto. Ituzaingó, Puerto Valle, 27°35′22.5″ S 56°31′34.2″ W, 82 m s.m., 21.05.2013, Keller et al. 11367.
27. *Trichomanes pellucens* Kunze, Depto. Ituzaingó, 12, 27°35′21.9″ S 56°31′35.6″ W, 25.02.2023, Meza Torres et al. 1747 (LIL).

LYGODIACEAE

28. *Lygodium volubile* Sw., Depto. Ituzaingó, Reserva Natural Santa Maria, 27°30′55.0″ S 56°40′03.0″ W, 83 m s.n.m., 27.07.2024, Villalba & Fariña 1.

LINDSAEACEAE

29. *Lindsaea divaricata* Klotzsch, Depto. Ituzaingó, Puerto Valle, 27°34′55.4″ S 56°31′50.5″ W, 83 m s.m., 24.05.2013, H. A. Keller et al. 11398.

MARCILEACEAE

30. *Marsilea ancylopoda* A. Braun, Depto. Mercedes, P. N. Iberá. Rincón del Socorro, 50 m s.n.m., 26.10.2017, Øllgaard & Meza Torres 3098.
31. *Regnellidium diphyllum* Lindm., Depto. Ituzaingó, 03.02.1982, Johnson 762.

OPHIOGLOSSACEAE

32. *Ophioglossum tuberosum* Hook. and Arn., Depto. Concepción, Paso Pucú, 64 m.s.n.m, 28°21′58.00″ S 57°55′9″ W, 12.09.2008, Meza Torres & Macluf 826.
33. *Ophioglossum nudicaule* L. f. Depto. Mercedes, entrada a Uguay, 28°42′41″ S 57°27′45″ W, 78 m s.m., 29.11.2009, Meza Torres & Klein 1242.
34. *Ophioglossum reticulatum* L. Dpto Mercedes. Macrosistema Ibera, Laguna Itatí, Isla Ombuzal, 28°42’ S, 58°06’ W, 27.08.1998, Schinini et al. 7982.

OSMUNDACEAE

35. *Osmundastrum cinnamomeum* (L.) C. Presl, Depto. San Martín, Costa W de la Laguna Iberá, 09.12.1992, Tressens et al. 4310.
36. *Osmunda spectabilis* Willd., Depto. Ituzaingó, Puerto Valle, 12.03.2016, Meza Torres et al. 1599.

POLYPODIACEAE

37. *Campyloneurum nitidum* (Kaulf.) C. Presl, Depto. Ituzaingó, Puerto Valle, Hogar de Niños Filadelfia, 27°34′24″ S 56°29′31″ W, 22.04.2007, Meza Torres et al. 611.
38. *Microgramma squamulosa* (Kaulf.) de la Sota, Mercedes, Ea. Culantrillar, Laguna Trin, 17.10.1975, Schinini 11680.
39. *Microgramma vacciniifolia* (Langsd. & Fisch.) Copel.,Bosques higrófilos y bosques con Prosopis. Depto. Ituzaingó, Laguna Isipo, Esteros del Iberá, 15.12.1976, Arbo et al. 1454.
40. *Pecluma pectinatiformis* (Lindm.) M.G. Price, Depto. Ituzaingó, Reserva Privada Pomera, 27°35′22.2″ S 56°31′34.5″ W, 29.05.2024, Polentarritti et al. 25.
41. *Pecluma robusta* (Fée) M. Kessler & A.R. Sm., Depto. San Martín, borde NO de la Laguna Iberá, costa Laurel-tí, 28°27′34″ S 57°7′33″ W, 74 m.s.n.m, 11.09.2008, Meza Torres et al. 824.
42. *Pleopeltis pleopeltifolia* (Raddi) Alston, Depto. Mercedes, Ayo. Pay Ubre Grande, 29°1′40″ S 58°10′30″ W, 80 m.s.m, 09.02.2008, Meza Torres et al. 704.
43. *Pleopeltis minima* (Bory) J. Prado & R.Y. Hirai. Depto. Mercedes, P. N. Iberá. Rincón del Socorro, 28°38′51.69″ S 57°24′57.23″ W, 50 m s.m., 26.10.2017, Øllgaard & Meza Torres 3091.

PSILOTACEAE

44. *Psilotum nudum* (L.) P. Beauv., Depto. Ituzaingó, 27°34′56.7″ S 56°31′49.,9″ W, 81 m s.m., 01.03.2013, Keller et al. 11316.

PTERIDACEAE

45. *Adiantopsis chlorophylla* (Sw.) Fée, Depto. Mercedes, P. N. Iberá, Rincón del Socorro, 50 m s.m., 26.10.2017, Øllgaard & Meza Torres 3082.
46. *Adiantopsis radiata* (L.) Fée, Depto. Ituzaingó, Puerto Valle, Hogar de los Niños de Filadelfia. 27°34′24″ S 56°29′31″ W, 22.04.2007, Meza Torres et al. 607.
47. *Adiantopsis tweediana* (Hook.) Link-Pérez & Hickey, Depto. Ituzaingó, Rincón de Santa María, 27°31′16″ S 56°36′05″ W, 66 m s.m., 27.04.2002, Keller et al. 14853.
48. *Adiantum lorentzii* Hieron., Depto. Mercedes, Ea. Culantrillar, 75 km N de Mercedes, Laguna Trin. 17.10.1975, Schinini et al. 11925.
49. *Adiantum raddianum* C. Presl, Depto. Mercedes, Macrosistema Iberá. Estancia Rincón del Diablo, 28.08.1998, Arbo 8019.
50. *Gastoniella chaerophylla* (Desv.) Li Bing Zhang & Liang Zhang, Depto. Mercedes, P. N. Iberá, Rincón del Socorro, 28°38′51.69″ S 57°24′57.23″ W, 50 m s.m., 26.10.2017, Øllgaard & Meza Torres 3105.
51. *Ceratopteris pteridoides* (Hook.) Hieron., Depto. Mercedes, Ea. Culantrillar, Laguna Trin, Isla El Disparo, 17.10.1975, Schinini et al. 12052.
52. *Doryopteris arifolia* Christ, Depto. Ituzaingó, Puerto Valle, 27°36′15″ S 56°30′49.4″ W, 21.05.2013, Keller et al. 11394.
53. *Doryopteris concolor* (Langsd. & Fisch.) Kuhn, Depto. Mercedes, P. N. Iberá, Rincón del Socorro, 28°38′51.69″ S 57°24′57.23″ W, 50 m s.m., 26.10.2017, Øllgaard & Meza Torres 3093.
54. *Doryopteris pentagona* Pic. Serm., Depto Ituzaingó, Puerto Valle, Hogar de los Niños de Filadelfia, 27°34′24″ S 56°29′31″ W, 22.10.2007, Meza Torres et al. 606.
55. *Doryopteris triphylla* (Lam.) Christ, Depto. Mercedes, Salto Ita Jhase, arroyo Ita Cora 18.08.1995, Tressens et al. 5332.
56. *Hemionitis tomentosa* (Lam.) Raddi, Depto. Mercedes, Macrosistema Iberá, nacimiento del rio Corriente, 27.08.1998, Arbo et al. 7957.
57. *Lytoneuron lomariaceum* (Klotzsch) Yesilyurt, Depto. Ituzaingó, Puerto Valle, 27°38′32.5″ S 56°30′34.4″ W, 83 m s.n.m., 31.07.2013, Keller et al. 11529.
58. *Pityrogramma calomelanos* (L.) Link var. *calomelanos*, Depto. Mercedes, P. N. Iberá, Rincón del Socorro, 28°38′51.69″ S 57°24′57.23″ W, 50 m s.m. 25.10.2017, Øllgaard & Meza Torres 3101.
59. *Pityrogramma trifoliata* (L.) R.M. Tryon, Depto. Ituzaingó, Área de Reclusión de Represa Hidroeléctrica Yaciretá, madrejón, 06.06.2016, Meza Torres et al. 1615.
60. *Vittaria lineata* (L.) Sm., Depto. Ituzaingó, Puerto Valle, 27°34′54.6″ S 56°31′52.6″ W, 72 m s.m., 24.05.2013, interior de bosque de Ararí en paleocauce del río Paraná, epífita en parte baja de tronco, Keller et al. 11404.

SALVINIACEAE

61. *Azolla cristata* Kaulf., Depto. Ituzaingó, Laguna Luna, 28°04′9.1″ S 56°49′50″ W, 24.11.1999, Arbo et al. 8493.
62. *Azolla filiculoides* Lam., Depto. Mercedes, Laguna Trin, Estancia Culantrillar, 17–24.10.1975, Schinini et al. 11827.
63. *Salvinia auriculata* Aubl. var. *major* C.V. Miranda & Schwartsb., Depto. Mercedes, Estancia Rincón del Diablo, Macrosistema Iberá, 28°42′ S 58°01′ W, 28.08.1998, Arbo et al. 7896.
64. *Salvinia biloba* Raddi, Depto. San Miguel, Laguna Po, 23.02.1976, Neiff 503.
65. *Salvinia minima* Baker, Depto. San Martín, Laguna Iberá, Cnia. C. Pellegrini, 05.11.1973, Arbo & Ferraro 415.
66. *Salvinia molesta* D.S. Mitch. Depto. San Martín, Iberá, 16.04.1980, Forno s.n. (Z).

ANEMIACEAE

67. *Anemia phyllitidis* (L.) Sw. var. *phyllitidis*, Depto. Mercedes, Salto Ita Jhase, 18.08.1995, Tressens et al. 5368.
68. *Anemia phyllitidis* (L.) Sw. var. tweedieana (Hook.) Hassl., Depto. Mercedes, Ayo. Pay Ubre Grande, 29°1′40″ S 58°10′30″ W, 80 m.s.n.m, 09.02.2008, Meza Torres et al. 705.
69. *Anemia tomentosa* (Savigny) Sw. var. *anthriscifolia* (Schrad.) Mickel, Depto. Ituzaingó, Reserva Natural Mbaracayá, 27°28′58.7″ S 56°42′17.0″ W, 73 m s.n.m., 11.07.2024, Cardinale & López 1.

THELYPTERIDACEAE

70. *Amauropelta rivularioides* (Fée) Salino & T.E. Almeida, Depto. San Martín, Carlos Pellegrini, 31.10.1971, Krapovickas et al. 20263.
71. *Cyclosorus interruptus* (Willd.) H. Itô, Depto. Concepción, Camino desde “El Tránsito″, 60 m s.m., s.f., Keller et al. 10913.
72. *Christella conspersa* (Schrad.) A. Löve & D. Löve, Depto. Ituzaingó, Ea. Santa María, 19.03.1982, Carnevali 5571.
73. *Christella dentata* (Forssk.) Brownsey & Jermy, Depto. Ituzaingó, Puerto Valle, 01.1990, Heinonen et al. 94.
74. *Christella hispidula* (Decne.) Holttum, Depto. Ituzaingó, 27°35′21.9″ S 56°31′35.6″ W, 25.02.2023, Meza Torres et al.1745 (LIL).
75. *Macrothelypteris torresiana* (Gaudich.) Ching, Depto. Ituzaingó, Puerto Valle, 27°35′22.5″ S 56°31′34.2″ W, 77 m s.m., 06.06.2013, Keller et al. 11412.
76. *Meniscium serratum* Cav., Depto. Santo Tomé, Laguna Galarza, Reserva Natural, 27.041995, Arbo et al. 6624.
